# Complex Presentation of Lung Cancer with Obstructive Jaundice

**DOI:** 10.3390/reports7020030

**Published:** 2024-04-24

**Authors:** Ruxandra Oprita, Bogdan Oprita, Ioana Adriana Serban, Lidia Aurelia Stefan, Ciprian Mihai Neacsu, Alice Elena Diaconu, Valentin Enache

**Affiliations:** 1 Faculty of Medicine, University of Medicine and Pharmacy “Carol Davila”, 050474 Bucharest, Romania; ruxandra.oprita@umfcd.ro (R.O.); bogdan.oprita@umfcd.ro (B.O.); 2 Gastroenterology Department, Clinical Emergency Hospital of Bucharest, 105402 Bucharest, Romania; lidiiastefan@yahoo.com (L.A.S.); neacsu.mihai.ciprian@gmail.com (C.M.N.); alice-elena.diaconu@rez.umfcd.ro (A.E.D.); 3 Emergency Department, Clinical Emergency Hospital of Bucharest, 105402 Bucharest, Romania; 4 Pathology Department, Clinical Emergency Hospital of Bucharest, 105402 Bucharest, Romania; valienache00@gmail.com

**Keywords:** SCLC, small-cell lung carcinoma, jaundice, abdominal pain, COPD, pancreatic metastasis

## Abstract

Background: Lung cancer, particularly small-cell lung carcinoma (SCLC), often presents with respiratory symptoms. However, atypical manifestations including jaundice and abdominal pain can obscure the diagnosis, leading to challenges in early detection and treatment. Case Presentation: A 49-year-old male, with a history of smoking and diagnosed with Chronic Obstructive Pulmonary Disease (COPD), presented to the emergency department with a 3-day history of jaundice and a 3-week duration of mild abdominal pain. Initial investigations, including blood tests, showed hyperbilirubinemia and elevated lipase and amylase levels. An abdominal ultrasound was performed and revealed a hypoechoic, inhomogeneous mass in the head of the pancreas and multiple liver masses, suggesting a cephalo-pancreatic formation with liver metastasis. Further diagnostic procedures, including upper endoscopy and ERCP, followed by a TAP CT scan, identified a large mediastinal-pulmonary mass with invasion into major vessels and extensive metastasis. The immunohistochemical analysis of a duodenal ulcer biopsy confirmed a diagnosis of duodenal metastasis from a small-cell neuroendocrine lung carcinoma. Conclusion: Our case highlights that while rare, the possibility of metastatic spread should be included in the differential diagnosis when obstructive jaundice occurs in the context of high-risk factors for lung cancer.

## 1. Introduction

Chronic Obstructive Pulmonary Disease (COPD), with a global prevalence rate between 7% and 19%, is the third leading cause of mortality worldwide. According to the World Health Organization’s (WHO) 2004 estimates, there are currently 64 million individuals suffering from Chronic Obstructive Pulmonary Disease (COPD), with 3 million deaths attributed to the condition. Also, COPD is an independent risk factor for lung cancer, with the prevalence of neoplasia being higher among patients diagnosed with COPD, especially those with low to mild respiratory dysfunction. Lung cancer is one of the most frequently diagnosed cancers worldwide; it is the second leading type of cancer (after prostate cancer) among men and, also, the second most frequently diagnosed cancer in women (after breast cancer). COPD associated with lung cancer contributes to increased morbidity and mortality.

This case study presents Patient Y, a 49-year-old male who presented to the Emergency Department with a three-day duration of jaundice and mild abdominal pain persisting for three weeks. Despite a history of Chronic Obstructive Pulmonary Disease (COPD) GOLD I and prior smoking, the patient was asymptomatic for respiratory issues at the time of presentation. The case unfolds a diagnostic journey that led to the identification of primary lung cancer with extensive metastasis, illustrating the critical importance of comprehensive diagnostic evaluations in patients with non-specific gastrointestinal symptoms and a history of smoking.

## 2. Detailed Case Description

Patient Y was admitted to the Emergency Room with a 3-day history of jaundice and a 3-week duration of mild, non-specific abdominal pain. Despite his COPD diagnosis GOLD I, mMRC Grade 0, SpO_2_ = 97%, he was asymptomatic in that regard at the time of presentation. His smoking history was also noted, marking him as at-risk for related complications.

### 2.1. Diagnostic Evaluation

Bloodwork indicated minimal leukocytosis, normal hemoglobin levels, marked hyperbilirubinemia, total bilirubin of 14 mg/dL (NV: 0.20–1.30 mg/dL), predominantly direct bilirubin 10 mg/dL (NV: 0.00–0.40 mg/dL), elevated lipase 1300 U/L (NV 23–300 U/L) and amylase 300 U/L (NV 30–110 U/L) levels, normal coagulation profile, and moderate hepatic cytolysis (subunitary deRitis ratio, AST 200 U/L (NV: 11–34 U/L), ALT 110 U/L (NV: 0–45 U/L), tumoral markers: CA 19-9 367 U/mL (NV: 0–37 U/mL) and CEA 56 ng/mL (NV: 0–3 ng/mL)).

The abdominal ultrasound revealed a dilated common bile duct (CBD) with a diameter of 12 mm, a hypoechoic, inhomogeneous solid mass with poorly defined borders in the head of the pancreas, and multiple similar masses in the liver.

The initial diagnosis leaned towards a cephalo-pancreatic mass with liver metastasis.

**Figure 1:**
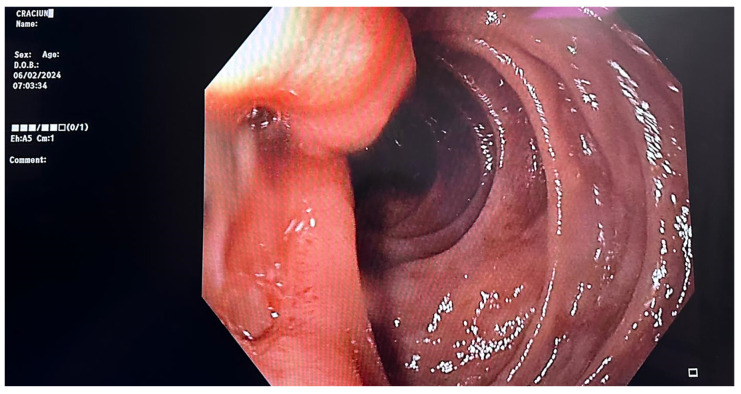
Duodenal ulcer distal to the papillary apparatus—endoscopic view.

Further investigations included an upper endoscopy, which unveiled normal gastric mucosa, a deformed pylorus due to edema, and in the second part of the duodenum, distal to the papillary apparatus, a 10 mm Forrest III ulcer with surrounding edema. Biopsies were obtained from the lesion (Figure 1).

The next step was performing an endoscopic retrograde cholangiopancreatography (ERCP). A guidewire-assisted cannulation of the common bile duct was performed, a precut sphincterotomy was carried out, then the CBD was cannulated, and a contrast substance was injected for diagnostic purposes. The mid-CBD stricture was visualized, with dilated CBD upstream (most likely by extrinsic compression), and it was decided to mount a 10 Fr/7 cm plastic biliary stent with effective drainage. Distal to the papillary apparatus, a malignant ulcer with deep crater and edematous raised edges was visualized, and multiple biopsies were taken.

**Figure 2:**
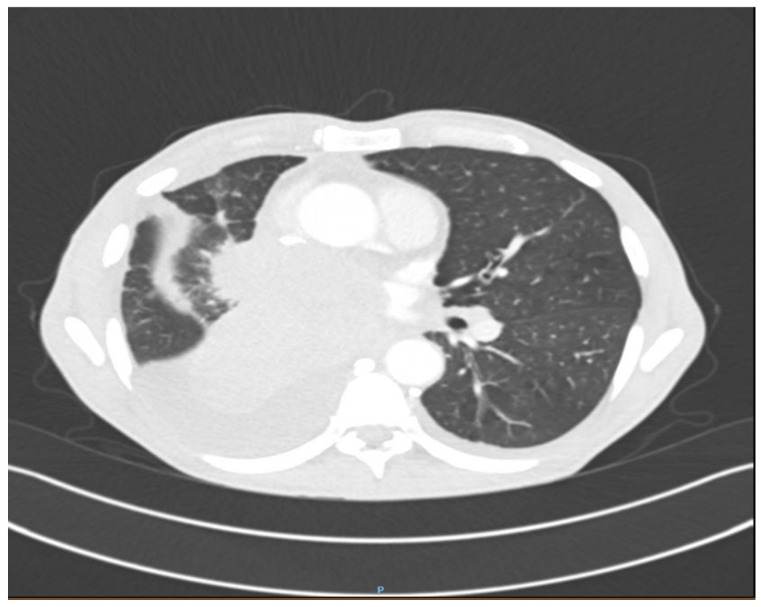
Right mediastinal pulmonary tumor process with vascular and cardiac invasion, associated with ipsilateral pleural effusion, with a most likely malignant substrate.

**Figure 3:**
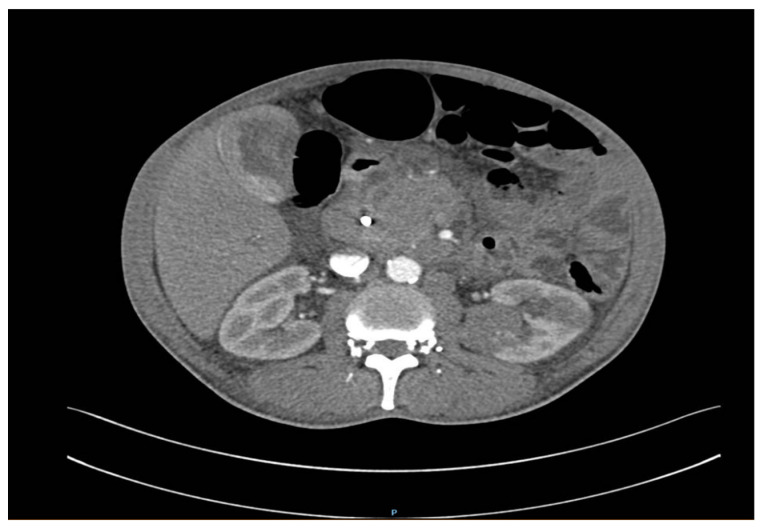
Diffuse tumor infiltrating the pancreas, associated with numerous adjacent peritoneal and retroperitoneal metastases. Renal metastases.

A CT thorax, abdomen, and pelvis scan with contrast on the following day revealed right pulmonary tumor formation, occupying the entire middle lobe (ML) and right inferior lobe (RIL), with dimensions of about 10/9/10 cm, non-homogeneous natively and postcontrast, with poly lobed contour, with an obstructive effect on the right pulmonary artery and the right middle and inferior bronchi. It extended mediastinally and invaded the terminal portions of the Superior Vena Cava (SVC) and Inferior Vena Cava (IVC), both right pulmonary veins, and the left atrium. (Figure 2). In addition, there were multiple pulmonary nodules in the right superior lobe with spiculiform contour, iodophilic with a retractile character over the adjacent parenchyma and pleura, and right pulmonary lymphangitis associated with a few peribronchovascular clustered micronodules with uncertain substrate up to 4 mm. Hepatomegaly was present with a cranio-caudal diameter of the right hepatic lobe of 20 cm regular contour. The structure of the liver was inhomogeneous due to the presence of hypodense formations with discrete peripheral iodophilia located in both liver lobes, the largest of which was a 10–12 mm segment II aspect of hepatic metastasis. A biliary stent was present and the cholecyst had no significant changes. The pancreas presented with diffuse tumorous infiltration, partially engulfed and surrounded by numerous isolated and confluent peritoneal and retroperitoneal metastases, causing the occlusion of the spleno-porto-mesenteric confluence, with the development of collateral circulation. Furthermore, bilateral adrenal and renal metastases, with diameters of approximately 5 cm, were present (Figure 3). The spleen was without CT changes, and no focal bone lesions suspicious of tumor substrate were detected.

**Figure 4:**
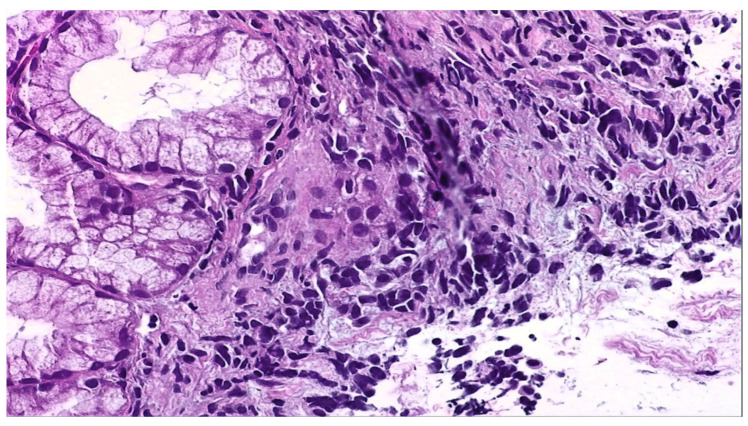
H&E stain 400×—Duodenal mucosa with monomorphic cells with hyperchromatic nuclei.

**Figure 5:**
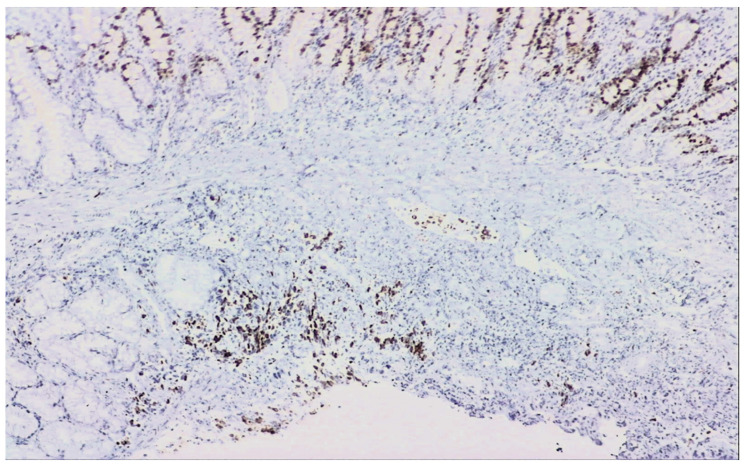
Ki67 positive 60% 100×—Sixty percent of the examined cancer cells are actively proliferating.

**Figure 6:**
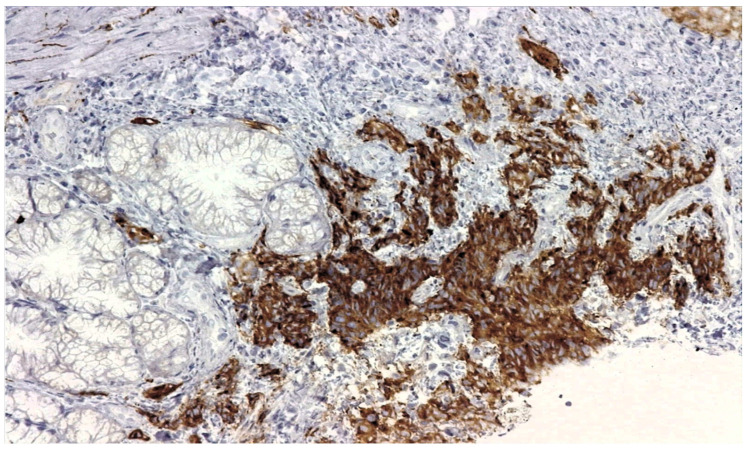
Synaptophysin (a protein found in the membranes of synaptic vesicles) positive 200×.

The biopsies taken from the duodenal ulcer distal to the papillary apparatus consisted of duodenal mucosa with preserved architecture and minimal lymphoplasmacytic infiltrate. On one of the fragments, there was an area of small–to medium-sized, monomorphic cells with hyperchromatic nuclei (Figure 4). Immunohistochemical tests: AE1 positive, TTF1 positive, CDX2 negative, LCA negative, and Ki67 positive 60% (Figure 5), synaptophysin positive (Figure 6). The histopathological aspects and immunohistochemical tests correlated with clinical data (lung tumor) supported the diagnosis of a duodenal metastasis of small-cell neuroendocrine lung carcinoma.

Upon clinical examination and subsequent paraclinical and imaging investigations, a final diagnosis was established indicating the presence of a right-sided mediastinal-pulmonary tumor with vascular and cardiac invasion. Additionally, secondary tumors were noted in the liver, pancreas, bilateral adrenal glands, kidneys, peritoneum, retroperitoneum, and the right pleura.

### 2.2. Management and Outcome

Given the extensive metastatic involvement and the primary tumor’s location, the patient was discharged clinically stable and referred to the oncology department for further management, emphasizing the need for specialized cancer care.

## 3. Discussion

According to the 2015 World Health Organization (WHO) classification of tumors of the lung, pleura, thymus, and heart, there are four distinct histologic variants of lung NETs: typical carcinoids (TCs), atypical carcinoids (ACs), large-cell neuroendocrine carcinoma (LCNEC), and small-cell lung carcinoma (SCLC). This classification helps in the differential diagnosis [1]. Neuroendocrine tumors represent around 20% of lung cancers, with SCLC accounting for the majority at around 14% [2]. SCLC is characterized by a rapid doubling time, high growth fraction, and the early development of widespread metastases. Most SCLC patients have hematogenous metastases, while approximately one-third of them have limited disease confined to the chest. SCLC is highly sensitive to initial chemotherapy and radiotherapy; however, most patients eventually succumb to recurrent disease [3]. Typically, SCLC presents as a large hilar mass and bulky mediastinal lymphadenopathy, causing cough and dyspnea [4]. Patients often have symptoms of widespread metastatic disease, including weight loss, debility, bone pain, and neurologic compromise.

Pancreatic metastasis from lung cancer, though common at autopsy, is rare in clinical settings. Pancreatic metastasis, which constitutes 2–5% of all pancreatic malignancies [5,6], is frequently derived from cancers of the lung, kidney, breast, skin (particularly melanoma), stomach, and large intestine [7,8]. Discussions on lung cancer spreading to the pancreas are scarce, yet small-cell lung carcinoma is more inclined to develop pancreatic metastasis compared to adenocarcinoma or squamous-cell carcinoma. This type of metastasis from small-cell lung carcinoma often presents as a single nodular lesion, closely resembling a primary pancreatic tumor [9]. In a study of 850 lung cancer patients, Maeno et al. found that 26 patients (3%) had pancreatic metastases, primarily presenting as a single nodule (73%), multiple nodules (11.5%), or diffuse swelling (15.4%). Additionally, a significant number of these patients also had liver (73.1%) and adrenal gland (69.2%) metastases. This highlights pancreatic metastasis as a notable site for extrathoracic disease spread in advanced lung cancer, particularly in small-cell lung cancer [10].

The manifestations of primary lung malignancy range from incidental detection in asymptomatic individuals to severe in the case of multiorgan metastasis. Jaundice is a rare and atypical sign of lung cancer. In the present case, the initial presentation with jaundice and abdominal pain leaned the diagnosis towards a hepato-biliary or pancreatic pathology. Furthermore, the ultrasound performed revealed a cephalo-pancreatic mass with bile duct obstruction and multiple hepatic metastasis. Taking into consideration the history of COPD and prior smoking, a CT TAP was performed, establishing the presence of a right-sided mediastinal-pulmonary tumor with vascular and cardiac invasion and multi-organ metastasis. The origin of the patient’s malignant duodenal ulcer was confirmed via cytology and immunohistochemistry. The biopsy tissue was stained for TTF-1, a marker indicative of primary lung origin, produced by type 2 alveolar pneumocytes involved in the synthesis of surfactant proteins. A total of 85% to 90% of SCLCs are positive for thyroid transcription factor-1 (TTF-1) [11]. In addition, the biopsy was AE1-positive. Nearly all SCLCs are immunoreactive for cytokeratin (AE1/Ae3, CAM5.2) [11].

This case reveals the complexity of diagnosing lung cancer, which may initially present with symptoms mimicking gastrointestinal or hepatobiliary diseases. The patient’s smoking history and COPD diagnosis were significant risk factors for lung cancer, which, in this case, manifested with jaundice and abdominal pain due to metastatic spread rather than respiratory symptoms.

## 4. Conclusions

Reported instances of pancreatic metastasis originating from primary lung cancer are rare within the medical literature. This case exemplifies the diagnostic challenges in patients with atypical symptoms and underscores the necessity of a thorough and multidisciplinary approach.

Our case highlights that while rare, the possibility of metastatic spread should be included in the differential diagnosis when obstructive jaundice occurs in the context of high-risk factors for lung cancer.

## Data Availability

The original contributions presented in the study are included in the article, further inquiries can be directed to the corresponding author.
